# Fibrosis-4 index is associated with the risk of hepatocellular carcinoma in patients with cirrhosis and nonalcoholic steatohepatitis

**DOI:** 10.3389/fonc.2023.1198871

**Published:** 2023-08-22

**Authors:** Somaya Albhaisi, Jing Sun, Arun J. Sanyal

**Affiliations:** ^1^ Department of Internal Medicine, Virginia Commonwealth University, Richmond, VA, United States; ^2^ Department of Epidemiology, Johns Hopkins Bloomberg School of Public Health, Baltimore, MD, United States; ^3^ Division of Gastroenterology, Hepatology and Nutrition, Department of Internal Medicine, Virginia Commonwealth University, Richmond, VA, United States

**Keywords:** Fibrosis-4 index (FIB-4), non-alcoholic steatohepatitis (NASH), cirrhosis, hepatocellular carcinoma (HCC), non-alcoholic fatty liver disease (NAFLD), outcome, noninvasive test

## Abstract

**Background and aims:**

Identification of high-risk patients for hepatocellular carcinoma (HCC) is essential for long term monitoring of nonalcoholic steatohepatitis (NASH) cirrhosis progression. We sought to evaluate the association between Fibrosis-4 (FIB-4) index and incidence of HCC risk among patients with NASH cirrhosis.

**Methods:**

We conducted a retrospective cohort study of adult patients with NASH cirrhosis (n= 1,338) who were evaluated in a single medical center between 2005 and 2015. Those who developed HCC were identified through electronic medical records using International Classification of Diseases (ICD) 9 and 10 codes until the end of September 2021.

**Results:**

During a median follow-up time of 3.7 years, 157 (11.7%) patients with NASH cirrhosis developed HCC. At index visit, the study population had a median age 57 years, 43% males, 78.8% White, and mean FIB-4 index 4.2. The final multivariable Cox regression model revealed that male sex, BMI 25-29.9 kg/m^2^, and hypertension were independent factors associated with development of HCC in patients with NASH cirrhosis. Compared to patients with FIB-4 ¾ 1.45, patients with FIB-4 between 1.45-3.25 had a similar hazard of HCC (Hazard Ratio [HR] 1.12, 95% CI: 0.67-1.86, p=0.670), whereas patients with FIB-4 >3.25 had a 1.93 (95% CI: 1.22-3.05, p=0.005) increased hazard of HCC.

**Conclusion:**

FIB-4 > 3.25 was an independent factor associated with increased HCC risk among NASH cirrhosis patients. FIB-4 index is a promising tool for determining high-risk patients and may be used in routine clinical practice to monitor risk of HCC in patients with NASH cirrhosis.

## Introduction

Cirrhosis, end-stage liver disease, hepatocellular carcinoma (HCC), and liver transplantation are all common outcomes of nonalcoholic fatty liver disease (NAFLD), which has emerged as a major global health issue (1). Nonalcoholic steatohepatitis (NASH), its progressive form, is the most common reason for liver transplantation in the United States, the fastest-growing cause of HCC in liver transplant candidates (2) and the most rapidly increasing indication for liver transplantation in young adults in the United States (3). Fibrosis stage is a strong predictor for disease progression and mortality in patients with NAFLD (4–6). Thus, early diagnosis of fibrosis is crucial to identify high-risk patients to improve outcomes. Liver biopsy is the gold standard to evaluate liver fibrosis but is not feasible for screening and disease monitoring in clinical practice due to its invasive nature, cost, risks, susceptibility to sampling errors, variable reliability and relatively low disease prevalence (7). Therefore, non-invasive methods or tests are essential for identifying patients with liver fibrosis and for long-term monitoring. One of the most studied non-invasive methods for estimating fibrosis stage and assessing liver stiffness is transient elastography. However, it is not widely available in all clinical settings, its use may be limited by increased body habitus, and it might not be a practical first-line strategy in primary care clinic settings or low-resource settings (8). Despite the fact that Liver Societies have the same screening recommendations (abdominal ultrasonography every six months with or without serum alpha-fetoprotein) in patients with cirrhosis irrespective of HCC risk (9–11), HCC surveillance is still underutilized in clinical practice, especially in patients with cirrhosis caused by NASH as many studies have reported poor compliance with these screening recommendations (12–15). Therefore, there remains a critical unmet need to develop and validate non-invasive biomarkers or tests that can be used for long-term monitoring of HCC in patients with cirrhosis during the everyday clinical practice, especially in low-resource settings (16). Numerous studies have been conducted to validate the diagnostic accuracy of non-invasive scoring systems based on clinical and biochemical markers for the diagnosis of liver fibrosis in NAFLD (8, 17, 18). The Fibrosis-4 index (FIB-4) is a non-invasive tool of readily available clinical parameters (age, aspartate aminotransferase [AST], alanine aminotransferase [ALT], and platelet count) that, when compared to other non-invasive fibrosis markers, has shown to be highly effective in identifying advanced fibrosis in patients with NAFLD (19, 20). The FIB-4 index has been demonstrated to have a prognostic value for predicting adverse liver-related outcomes in NAFLD patients (20). In general, a value of FIB-4 < 1.45 was the cut-off to rule-out cirrhosis, while a value of FIB-4 > 3.25 showed a high specificity for ruling in cirrhosis. Recent evidence suggests that FIB-4 index can predict adverse outcomes in NAFLD patients (21–24) but more studies in different populations and settings are needed to confirm its prognostic value. Therefore, the aim of this study was to assess the value of FIB-4 index in predicting the risk of HCC in a population with NASH cirrhosis.

## Patients and methods

### Study design

In this retrospective cohort study, we identified all adult patients (age ≥ 18 years) with NASH-cirrhosis who received inpatient and outpatient care in Virginia Commonwealth University Health System (VCUHS) between January 2005 and December 2015 (n =1,338). We used baseline characteristics in the initial visit to identify factors that may predict the risk of HCC in NASH cirrhosis. Study researchers made the determination that this study does not constitute human subject research given that the study uses secondary de-identified data (25).

### Patient population and data source

We derived data on all patients with NASH cirrhosis from electronic medical records (CERNER). Patients with NASH cirrhosis were identified using International Classification of Diseases (ICD) 9 and 10 diagnosis codes and billing data. Data extracted from patients’ records included demographic data (age, sex, race, ethnicity), smoking status, lab tests (complete blood count (white blood cell, hemoglobin, platelet counts), hepatic panel (ALT, AST, total bilirubin, direct bilirubin, albumin, and international normalized ratio (INR)), kidney function (creatinine, glomerular filtration rate (GFR)), lipid panel (total cholesterol, low-density lipoprotein (LDL), high-density lipoprotein (HDL), triglycerides), hemoglobin A1C (HbA1C)), comorbidities (type 2 diabetes mellitus (T2DM), hypertension, coronary artery disease, heart failure, sleep apnea, other cancers), and vital signs. We excluded patients with an HCC diagnosis documented prior to index date for NASH cirrhosis diagnosis, or had other etiologies of chronic liver disease including viral, alcoholic, cholestatic, autoimmune or genetic (**Supplementary Table 1**), or who received a liver transplant prior to the start of the study. In the absence of alcohol use disorders and ICD diagnostic codes for other cirrhosis etiologies (**Supplementary Figure 1**), the diagnosis of NASH cirrhosis was based on the presence of the ICD 9 and 10 codes for cirrhosis due to NAFLD or NASH (571.5, 571.8, 571.9, K75.81, K76.0), recorded at least once in any inpatient or outpatient encounter. We identified 1,338 individuals with a NASH cirrhosis diagnosis that was initially reported at or before 1/1/2005 and who underwent medical treatment at VCUHS between 2005 and 2015, determined by having at least one inpatient or outpatient visit for any indication during the study period.

### Clinical characteristics and outcomes of interest

Comorbidities were identified using ICD-9 and 10 codes. The patients’ baseline characteristics were ascertained at the index visit (first encounter) when a diagnosis of NASH cirrhosis was first recorded. Body mass index (BMI) was calculated as weight in kilograms divided by the square of height in meters. Patients were categorized based on their BMI as follows: underweight (BMI < 18.5 kg/m2), normal weight (BMI 18.5-24.9 kg/m2), overweight (BMI 25-29.9 kg/m2), and obese (BMI ≥ 30 kg/m2). We obtained results closest to index date for patients who had multiple lab tests or measurements. The diagnosis of HCC was based on the presence of ICD-9 codes (155, 155.0, 155.1) and ICD-10 (C22.0, C22.1) codes recorded at least once during the study follow up. FIB-4 index was calculated using standard formula which includes age, AST (U/L), ALT (U/L), and platelet count (10^9^/L) (26). Based on FIB-4 values, patients were categorized as (1) low risk, FIB-4 < 1.45; (2) intermediate risk, FIB4 1.45-3.25; and (3) high risk, FIB-4 > 3.25 (26–28). The primary outcome was HCC which was evaluated utilizing ICD codes that occurred during follow-up and were at least 90 days past index date.

### Statistical analysis

**Figure 1:**
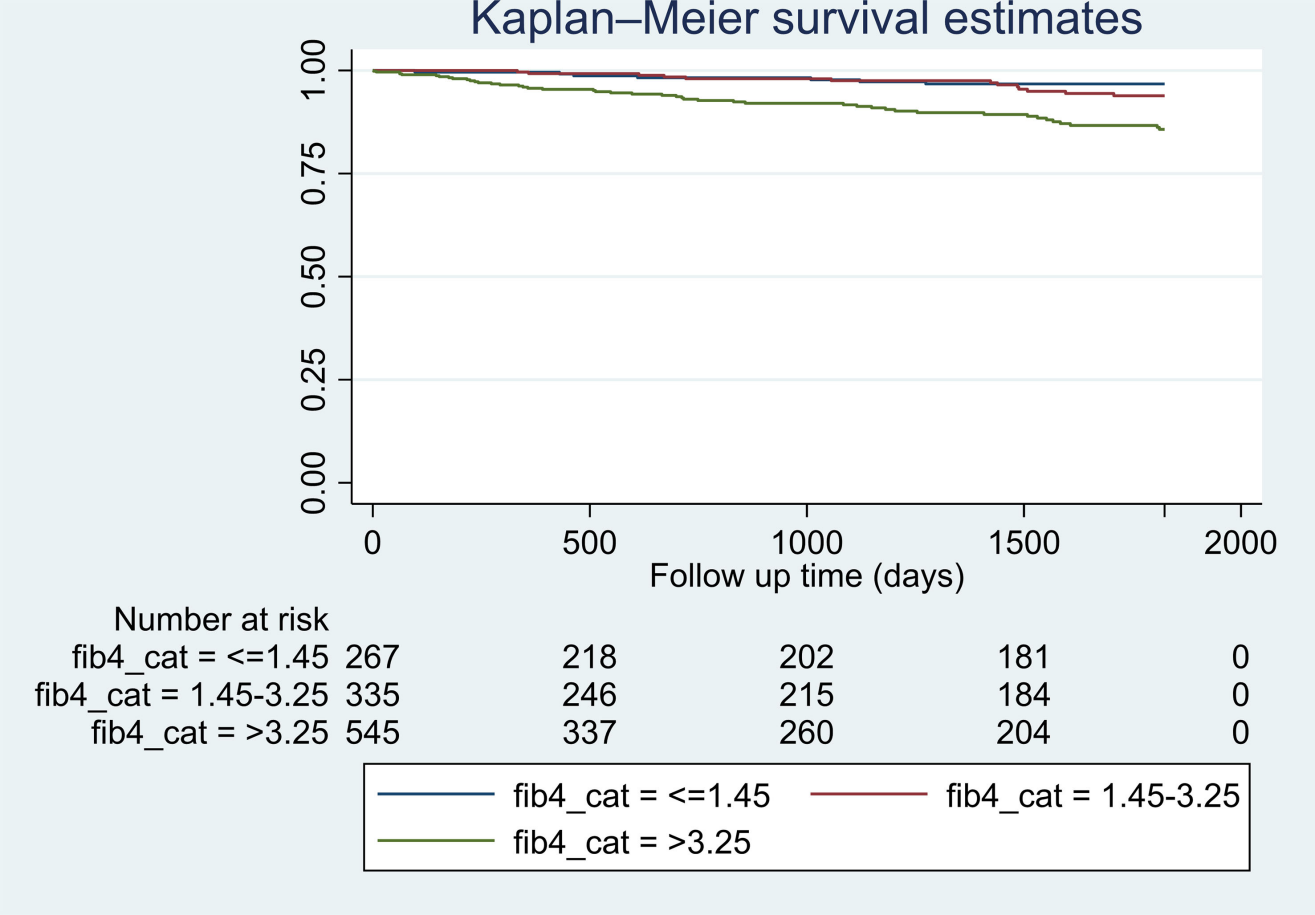
Kaplan-Meier estimates of hazard of developing HCC in patients with NASH cirrhosis by categories of FIB-4 index within 5 years of follow up. *Log-rank test statistic p-value <0.001.

Descriptive statistics were calculated to investigate sample characteristics. Continuous variables were summarized as median (interquartile range [IQR]) and mean (± standard deviation [SD]) and categorical variables as frequency (percentage). Follow-up time was defined as the number of years from the first diagnosis of cirrhosis (index date) to the diagnosis of HCC or to death, or until censoring due to no events at end of study. Follow up started at index date which could be as early as 2005 and continued until 9/30/2021, and it was used to calculate person-time at risk for HCC. Patients who did not develop HCC by 9/30/2021 were censored at the time of death or administratively censored at the end of study. Time to HCC analysis was performed using univariate and multivariable Cox proportional models, adjusting for factors of interest. In the final analyses and for the purposes of this study, we estimated the risk of HCC development within 5 years of follow up. Kaplan-Meier curves were used to illustrate time from baseline (index date) to HCC by FIB-4 index category (**Figure 1**). Univariate and multivariate Cox proportional hazard regression analyses were used to evaluate associations between patient characteristics and outcome, and to examine the associations between HCC and factors of interest, respectively. Cox regression results were reported as hazard ratio (HR) and 95% confidence interval (95% CI). Both Wald and likelihood ratio methods were used to test for the statistical significance of covariates in the final multiple proportional hazards model. Models were adjusted for sex, race/ethnicity, number of comorbidities, and laboratory values. We evaluated multiple multivariable models. The 3 final models were (a) Model 1 (main model): adjusted for sex, race/ethnicity, BMI, FIB-4 index, T2DM and hypertension; (b) Model 2: adjusted for age, liver-related lab tests in addition to other variables from model 1 but without FIB-4 index; (c) Model 3: adjusted for all aforementioned variables except age and platelet count since they are accounted for in the FIB-4 index. Model 1 was not adjusted for age or liver-related lab tests (platelet count, INR, albumin, total bilirubin) to avoid overadjustment since age and platelet count are included in the FIB-4 index calculation, and strongly correlated with liver-related labs and FIB-4 index. Model 2 evaluates the liver-related labs in absence of FIB-4 index. Model 3 assesses the possible correlation between FIB-4 index and liver-related labs (INR, albumin, total bilirubin). A sensitivity analysis following single imputation of mean for missing BMI values was performed due to a large number of missing values in BMI measurement. A two-tailed *P* value < 0.05 was considered significant. STATA version 17 software (StataCorp. 2021. Stata Statistical Software: Release 17. College Station, TX: StataCorp LLC) was used for all analyses.

## Results

### Baseline characteristics of study population

A flowchart for selection of study population is shown in **Supplementary Figure 1**. Baseline characteristics are summarized in **Table 1**. Overall, 43% of patients were males and 78.8% were non-Hispanic White. The entire population had a median age 57 (IQR 16) years. 26.2% had T2DM, 33.3% had hypertension, 2.4% had prior myocardial infarction, 9.4% heart failure, and 13.8% other cancers. 9.4% of patients had model for end-stage liver disease (MELD) score > 20. The proportion of patients with FIB-4 >3.25 was 46.7%. Patients with FIB4 >3.25 were more likely to be older (median age 60 years), males (50%), non-Hispanic White (82%), and have higher cholesterol ≥200 mg/dl (35%) (**Table 1**).

**Table 1:** Baseline characteristics of study population (overall and by FIB-4 index category).

	Overall (N = 1,338)	FIB-4 <=1.45 (N = 282)	FIB-4 1.45-3.25 (N = 352)	FIB-4 >3.25 (N = 556)
**Age, median (IQR), y**	57 (48-64)	48 (39-57)	58 (48-64)	60 (54-66.5)
**Male (n (%))**	574 (42.9)	105 (37)	139 (39)	277 (50)
Race/Ethnicity (n (%))				
White, non-Hispanic	1,045 (78.8)	199 (71)	279 (79)	445 (82)
Black, non-Hispanic	217 (16.4)	67 (24)	62 (18)	72 (13)
Hispanic	36 (2.7)	8 (3)	5 (1)	16 (3)
Other	29 (2.2)	8 (3)	5 (1)	13 (2)
**BMI, median (IQR), Kg/m2 ^+^**	31.9 (26.9-37.6)	32.9 (26.2-38.9)	31.2 (27.1-36.3)	31.1 (26.6-37.1)
**Diabetes mellitus (n (%))**	351 (26.2)	87 (31)	97 (28)	129 (23)
Insulin use	129 (9.6)	32 (11.3)	30 (8.5)	64 (11.5)
**Hypertension (n (%))**	445 (33.3)	126 (45)	130 (37)	141 (25)
Cardiac disease (n (%))				
Prior MI	32 (2.4)	8 (2.8)	10 (2.8)	14 (2.5)
CHF	126 (9.4)	39 (14)	34 (9.7)	47 (8.5)
Smoking (n (%)) *				
Never	417 (31.2)	99 (35)	105 (30)	171 (31)
Current	132 (9.9)	40 (14)	35 (10)	44 (8)
Former smoker	205 (15.3)	36 (13)	58 (16)	85 (15)
**Other cancers (n (%))**	184 (13.8)	46 (16)	60 (17)	63 (11)
Laboratory Results, (mean ± SD)				
MELD score **	8.2 (8.5)	4.3 (7.4)	6.03 (6.6)	11.3 (8.9)
Hemoglobin (g/dL) **	10.6 (2.7)	11.3 (3.0)	10.6 (2.5)	9.99 (2.5)
Platelet Count (x10^9^/L) **	179.1 (101.0)	291.2 (98.1)	196.6 (63.0)	109.8 (51.9)
Creatinine (mg/dL) **	1.2 (1.3)	1.3 (1.7)	1.1 (1.3)	1.2 (0.97)
Total cholesterol (mg/dl) **	164.9 (59.7)	173.1 (51.9)	170.5 (58.7)	154.7 (65.3)
LDL (mg/dl) **	94.6 (43.3)	96.3 (39.3)	98.9 (45.9)	90.2 (44.2)
Triglycerides (mg/dl) **	160.9 (140.3)	181.8 (133.6)	158.9 (96.6)	150.9 (172.8)
ALT (U/L) **	70.3 (239.0)	57.8 (52.1)	63.4 (84.8)	81.7 (344.1)
AST (U/L) **	73.8 (160.7)	42.7 (28.6)	60.0 (54.2)	99.1 (230.0)
ALP (U/L) **	167.4 (172.7)	161.8 (174.9)	163.2 (177.5)	173.8 (170.4)
Bilirubin, total (mg/dL) **	1.9 (4.0)	1.0 (2.8)	1.1 (2.1)	2.9 (5.1)
INR **	1.3 (0.47)	1.1 (0.3)	1.2 (0.3)	1.4 (0.6)
Albumin (g/dL) **	3.7 (0.73)	4.1 (0.7)	3.8 (0.7)	3.4 (0.6)
Hemoglobin A1c (%) **	6.7 (1.7)	6.8 (1.7)	6.7 (1.8)	6.5 (1.6)
Proportions with abnormal lab results (n (%))				
** Liver enzymes (U/L)** ALT ≥ 30 AST ≥ 30	876 (72.3) 1,014 (83.5)	200 (70.9) 169 (59.9)	247 (70.1) 306 (86.9)	412 (74.1) 519 (93.3)
** Total cholesterol (mg/dL)** ≥ 200	89 (30.8)	24 (28)	30 (30)	34 (35)
** LDL cholesterol (mg/dL)** 100-130 ≥ 130	144 (40.9) 59 (16.8)	43 (15) 17 (18)	48 (14) 17 (15)	50 (9) 24 (18)
** MELD score** 20-29 30-39 ≥ 40	73 (6.6) 25 (2.3) 6 (0.54)	12 (4.3) 1 (0.4) 0 (0)	15 (4.3) 2 (0.6) 0 (0)	45 (8.1) 21 (3.8) 6 (0)
** FIB-4 score** ¾1.45 1.45-3.25 >3.25	282 (21.1) 352 (29.6) 556 (46.7)			

ALT, alanine aminotransferase; AST, aspartate aminotransferase; ALP, alkaline phosphatase; BMI, body mass index; CHF; congestive heart failure; FIB-4, fibrosis-4; HCC; hepatocellular carcinoma; INR, international normalized ratio; IQR, interquartile range; LDL, low density lipoprotein; MELD, model for end-stage liver disease; MI, myocardial infarction; SD, standard deviation.

+Missing values in 432 patients.

*Missing data in 584 patients.

**Missing values in 221, 1,142, 121, 150, 958, 986, 966, 126, 123, 124, 128, 156, 126, 887, 148 patients, respectively.

### Univariate and multivariable associations with HCC

The median follow-up time was 3.7 years (IQR: 0.7-7.9 years). Among 1,338 patients with NASH cirrhosis, 157 (11.7%) developed HCC. The crude incidence rates for HCC, stratified by FIB-4 category, were 0.020, 0.024, and 0.052 per person-year, respectively, in patients with FIB-4 ¾1.45, 1.45-3.25, and >3.25. In the univariate analysis, factors that were identified to be significant predictors of the risk of HCC in NASH cirrhosis were older age (> 69 years), male sex, overweight/obesity, T2DM, hypertension, FIB-4 >3.25, albumin, INR, total bilirubin, and platelet count (**Table 2**). We tested multiple models to evaluate for possible confounding between FIB-4 index and liver-related laboratory values. In multivariable analyses, with adjustment for factors of interest based on univariate analysis, factors that maintained a significant association with HCC in NASH cirrhosis included (a) male sex, BMI 25-29.9 kg/m^2^, hypertension, and FIB-4 >3.25 in model 1.; (b) age >69 years, male sex, BMI 25-29.9 kg/m^2^, BMI 30-34.9 kg/m^2^, BMI 35-39.9 kg/m^2^, hypertension, and albumin in model 2; (c) male sex, BMI 25-29.9 kg/m^2^, BMI 30-34.9 kg/m^2^, BMI 35-39.9 kg/m^2^, hypertension, and albumin in model 3 (**Table 2**). Due to a large number of missing BMI values, we performed single value imputation and sensitivity analysis for the BMI variable and results were consistent between the original model and the sensitivity analysis model (**Supplementary Table 2**). FIB-4 >3.25 was significantly associated with HCC in the univariate analysis (HR 2.65, 95% CI: 1.71-4.13, p=0.000). In multivariate analysis of the main final model (model 1), compared to patients with FIB-4 ¾ 1.45, patients with FIB-4 between 1.45-3.25 had a similar risk of HCC (HR 1.12, 95% CI: 0.67-1.86, p=0.670), whereas patients with FIB-4 >3.25 had a 1.93 (95% CI: 1.22-3.05, p=0.005) increased hazard of HCC (**Table 2**).

**Table 2:** Factors Associated with HCC in NASH Cirrhosis Among Patients Evaluated in a Single U.S. Center Between 2005-2015.

	Univariate		Multivariable					
Factors	Unadjusted HR (95% CI)	p-value	Adjusted HR (95% CI)	p-value	Adjusted HR (95% CI)	p-value	Adjusted HR (95% CI)	p-value
			Model 1^+^		Model 2		Model 3	
FIB-4 score								
<1.45	1 [reference]		1 [reference]				1 [reference]	
1.45-3.25	1.31 (0.79-2.18)	0.296	1.12 (0.67-1.86)	0.670			1.02 (0.61-1.72)	0.936
>3.25	2.65 (1.71-4.13)	0.000	1.93 (1.22-3.05)	0.005			1.47 (0.89-2.45)	0.135
Age group, yrs								
¾49	1 [reference]				1 [reference]			
>49-59	1.21 (0.79-1.85)	0.374			1.09 (0.69-1.71)	0.719		
>59-69	1.46 (0.96-2.24)	0.078			1.32 (0.84-2.07)	0.230		
>69	2.23 (1.26-3.97)	0.006			2.20 (1.15-4.19)	0.017		
Sex								
Female	1 [reference]		1 [reference]		1 [reference]		1 [reference]	
Male	2.35 (1.70-3.24)	0.000	2.25 (1.59-3.19)	0.000	2.49 (1.74-3.57)	0.000	2.29 (1.60-3.26)	0.000
Race and ethnicity								
Hispanic	0.93 (0.34-2.50)	0.878	1.04 (0.33-3.32)	0.940	1.12 (0.35-3.62)	0.851	1.16 (0.36-3.72)	0.799
Non-Hispanic								
White	1 [reference]		1 [reference]		1 [reference]		1 [reference]	
Black	0.78 (0.50-1.20)	0.254	1.02 (0.64-1.62)	0.935	0.92 (0.57-1.50)	0.748	0.89 (0.55-1.43)	0.628
Other*	0.28 (0.04-2.02)	0.208	0.21 (0.03-1.49)	0.118	0.19 (0.03-1.34)	0.095	0.19 (0.03-1.40)	0.103
**BMI, Kg/m2**	0.976 (0.95-1.00)	0.063						
BMI category, Kg/m2								
<18.5	1.27 (0.16-10.15)	0.822	1.77 (0.22-14.44)	0.595	2.03 (0.25-16.67)	0.511	1.79 (0.22-14.63)	0.587
18.5–24.9	1 [reference]		1 [reference]		1 [reference]		1 [reference]	
25.0–29.9	2.90 (1.36-6.19)	0.006	3.12 (1.44-6.76)	0.004	3.44 (1.57-7.55)	0.002	3.51 (1.60-7.70)	0.002
30.0–34.9	2.18 (1.05-4.51)	0.036	2.07 (0.98-4.37)	0.057	2.61 (1.21-5.61)	0.014	2.49 (1.16-5.35)	0.019
35.0–39.9	1.70 (0.74-3.88)	0.208	1.98 (0.84-4.64)	0.117	2.89 (1.21-6.92)	0.017	2.57 (1.07-6.17)	0.034
≥40	0.90 (0.36-2.28)	0.827	1.00 (0.35-2.85)	0.998	1.24 (0.43-3.56)	0.689	1.15 (0.40-3.31)	0.794
**Diabetes**	0.60 (0.40-0.90)	0.014	1.09 (0.66-1.80)	0.745	1.03 (0.6301.70)	0.893	1.05 (0.63-1.74)	0.852
**Hypertension**	0.49 (0.34-0.72)	0.000	0.54 (0.34-0.87)	0.011	0.53 (0.33-0.85)	0.009	0.59 (0.37-0.94)	0.028
MELD score								
¾20	1 [reference]							
>20	1.10 (0.54-2.24)	0.794						
**Albumin, g/dL**	0.602 (0.48-0.75)	0.000			0.59 (0.45-0.78)	0.000	0.65 (0.48-0.88)	0.005
**INR**	1.40 (1.02-1.92)	0.038			1.05 (0.62-1.76)	0.864	0.97 (0.56-1.67)	0.907
**Creatinine, mg/dL**	0.84 (0.66-1.08)	0.170						
**Total Bilirubin, mg/dL**	1.03 (1.00-1.07)	0.031			0.99 (0.95-1.04)	0.804	0.99 (0.95-1.03)	0.620
**Platelet count, x10^9^/L**	0.996 (0.994-0.998)	0.000			1.00 (0.998-1.00)	0.924		

+Main final model.

*Other category included Asian, American Indian-Alaskan or other.

## Discussion

In a cohort of high risk NASH cirrhosis patients, we observed that FIB-4 index above 3.25 is an independent risk factor for HCC within a 5-year follow-up period. Taking into account the challenges of performing liver biopsies in all NAFLD patients as well as the risks associated with the procedure, the FIB-4 index can be a good clinical tool that is simple and easy to use for care of NAFLD patients (29). Our observations are in line with previous studies (22, 23, 30, 31) which reported that high FIB-4 is linked to a higher risk of adverse liver-related outcomes including HCC. Studies that evaluated predictors of HCC in patients with NAFLD, similar to our study, also showed that high risk FIB-4 scores were linked to an elevated risk of HCC and may be useful in predicting the development of hepatic and extra-hepatic malignancies (32, 33). Therefore, FIB-4 can be used as a non-invasive tool to identify patients at risk of developing HCC and to stratify those at high risk who could benefit from intensive treatment, closer monitoring and inclusion in clinical trials (30). This is particularly important in low-resource settings and where resources for HCC surveillance and routine screening are limited. Since several studies have suggested that FIB-4 score could predict HCC in the general population and among people with NAFLD/NASH, our study is adding a much needed validation of this association to the literature in order to translate this to clinical practice and to improve quality of care. Health care settings that do not have resources for liver services or lack access to Hepatology providers would benefit from studies like ours such that any health care provider can use FIB-4 index for long term monitoring of disease progression and potentially screening of high-risk groups to improve clinical outcomes. In addition, non-Hepatologists can use the FIB-4 index as a guide to implement the HCC screening recommendations by following established guidelines, or refer patients with high FIB-4 scores to Hepatologists.

Our data provide additional proof for the use of the FIB-4 index as a follow-up approach to monitor for the risk of development of HCC, firmly supporting the European practice guidelines on non-invasive testing for evaluation of liver disease severity and prognosis (34, 35). The FIB-4 index is comparable to other liver-related measurements (e.g. albumin, INR) given that it is based on the results of AST level, ALT level, and platelet count. Furthermore, age, AST, ALT, and platelet count are known to be associated with liver fibrosis (36–38). The strong correlation between the FIB-4 index and other liver-related measurements likely explains why the FIB-4 index was not a significant factor in the multivariable models that accounted for other liver-related measurements and therefore FIB-4 is not independent of these metrics.

The explanation for the association between HCC and FIB-4 index observed in our study can be found in previous literature. Liver fibrosis and cirrhosis are well-known risk factors for the development of HCC (29, 39, 40) and given that the FIB-4 index has been validated for the evaluation of liver fibrosis, we hypothesized that the FIB-4 index can predict the risk of developing HCC in patients with NASH cirrhosis. A low FIB-4 index was shown to have a considerably reduced risk of HCC than a high FIB-4 index, even in NAFLD patients without cirrhosis (cutoff value = 2.67) (41). Proposed mechanisms for development of HCC in NAFLD include insulin resistance, oxidative stress, lipotoxicity, and altered gut microbiome (42–44). Interestingly, a considerable proportion of patients in our study population had low risk FIB-4 scores ¾1.45. This could be related to the modest predictive ability of non-invasive scoring systems like FIB-4 in evaluating fibrosis (19, 45, 46).

In our cohort, most patients with NASH cirrhosis who developed HCC were older (age >69 years), men, and had BMI ≥25 kg/m^2^. These findings are consistent with previous studies (47). Surprisingly, T2DM was not significantly associated with risk of HCC and patients who had hypertension were at lower risk for development of HCC contrary to what has been reported in previous studies. This could be related to many factors and therefore still requires further investigation. For the purpose of this study, we considered T2DM and hypertension confounding factors for this association.

Our study has several strengths, including a relatively large patient population, combining data from all health care settings (inpatient, outpatient), and reliable baseline data. On the other hand, there are several limitations. It is a single center study, which may limit the generalizability of the findings. Diagnoses were made using ICD coding, which could result in underestimation of NAFLD due to misclassification bias. The other major limitation is the outcome HCC definition. Since this is a single center study, patients may have entered the study as NASH diagnosis but were then diagnosed with HCC in another medical center or a different setting so these cases were not captured in our data. ICD codes for NAFLD/NASH lack accuracy, and depend on the quality of data collection which can lead to coding errors. Given that we do not know whether the NASH diagnosis was actually made based on histology data, the associations should be carefully evaluated. We were unable to confirm fibrosis using imaging or liver biopsy to more accurately characterize the severity of liver disease across patients. In addition, we did not have information about NAFLD treatment modalities the patients may have received, which could affect their risk for HCC. Although we excluded alcohol related cirrhosis, patients might still consume alcohol. Given the nature of electronic health records’ database, certain measurements like waist circumference and laboratory values including fasting blood glucose are not available in the study population. Due to the significant number of missing BMI values in the statistical analysis, we applied imputation techniques for the missing BMI data. The results were consistent, which is considered a positive indicator for robustness of the data. Given the potential confounding effects of obesity, we controlled for BMI in our final model. Our findings show that the independent association between the FIB-4 index and HCC was not confounded by the BMI, which makes it unlikely that the relationship between the two variables can be explained solely by the BMI.

## Conclusion

In conclusion, a high FIB-4 score (>3.25) was strongly associated with risk of HCC in patients with NASH cirrhosis, independent of traditional clinical factors. These findings indicate that advanced fibrosis stages are strong predictors of HCC. FIB-4 index is a non-invasive, inexpensive, readily available in most clinical settings, and sensitive marker that can identify patients at risk of HCC and should be used for long-term monitoring, and potentially screening, of high-risk groups to improve clinical outcomes. Providers should focus on aggressive treatments for patients with severe liver fibrosis and pay close attention to FIB-4 index for long-term monitoring of the risk of developing HCC. Individuals with high FIB-4 index should have ultrasound monitoring in accordance with guidelines recommendations.

## Data availability statement

The raw data supporting the conclusions of this article will be made available by the authors, without undue reservation.

## Ethics statement

Ethical approval was not required for the study involving humans in accordance with the local legislation and institutional requirements. Written informed consent to participate in this study was not required from the participants or the participants’ legal guardians/next of kin in accordance with the national legislation and the institutional requirements.

## Author contributions

Conceptualization, SA, JS and AS. Methodology, SA, JS and AS. Software, SA and JS. Validation, SA, JS and AS. Formal Analysis, SA and JS. Investigation, SA, JS and AS. Resources, SA and AS. Data Curation, SA and AS. Writing – Original Draft Preparation, SA. Writing – Review & Editing, SA, JS and AS. Visualization, SA, JS and AS. Supervision, JS and AS. Project Administration, SA. Funding Acquisition, JS and AS. All authors contributed to the article and approved the submitted version.
