# Supplementary material for: Fibrosis-4 index is associated with the risk of hepatocellular carcinoma in patients with cirrhosis and nonalcoholic steatohepatitis
Source: Front Oncol. 2023 Aug 22;13:1198871. doi: 10.3389/fonc.2023.1198871 (PMC10477779; doi:10.3389/fonc.2023.1198871)
Supplement: Supplementary file 1 [file DataSheet_1.pdf]

SUPPLEMENTARY MATERIAL

Supplementary Table 1: ICD 9 and 10 codes for NAFLD/NASH and exclusionary diagnoses.

Inclusion:
571.8 other chronic nonalcoholic liver disease
571.9 unspecified chronic liver disease without alcohol
571.5 - Cirrhosis of liver without mention of alcohol
K75.81 nonalcoholic steatohepatitis
K76.0 fatty liver, NOS

Exclusion:
571.0 alcoholic fatty liver disease
571.1 acute alcoholic hepatitis
571.2 alcoholic cirrhosis of liver
571.3 alcoholic liver damage
K70.10-11 alcoholic hepatitis/liver disease
F10 alcohol dependence/abuse
070.0-9 viral hepatitis A, B, C, D, E and unspecified
V02.60-69 carrier or suspected carrier of viral hepatitis
571.40-41 chronic hepatitis unspecified
571.42 autoimmune hepatitis
571.49 chronic hepatitis
573.1-2 hepatitis in viral diseases classified elsewhere
B15.0-B19.9 Hepatitis A, B and C
Z22.50-59 Carrier of viral hepatitis
K71.2-6 Toxic liver disease
K73.0-9 Chronic hepatitis
K75.2-4 Autoimmune hepatitis
O98 Viral hepatitis complicating pregnancy
275.01 hemochromatosis
E83.11 hemochromatosis
E88.01 Alpha-1-antitrypsin deficiency
E83.01 Wilson’s disease

Supplementary Table 2: Sensitivity analysis following imputation for missing BMI values.

Factors	Multivariable Adjusted Hazard Ratio (95% Confidence Interval)	p-value
**Sex**		
Female	1 [reference]	
Male	2.21 (1.56-3.12)	0.000
**Race and ethnicity**		
Hispanic	0.96 (0.30-3.04)	0.945
Non-Hispanic		
White	1 [reference]	
Black	0.95 (0.60-1.50)	0.826
Other*	0.21 (0.03-1.50)	0.119
**BMI, Kg/m2**		
**BMI category, Kg/m2**		
<18.5	1.45 (0.18-11.8)	0.727
18.5–24.9	1 [reference]	
25.0–29.9	3.04 (1.41-6.57)	0.005
30.0–34.9	2.13 (1.01-4.50)	0.047
35.0–39.9	1.74 (0.75-4.05)	0.197
≥40	0.93 (0.33-2.60)	0.891
**FIB-4 score**		
<1.45	1 [reference]	
1.45-3.25	1.17 (0.70-1.94)	0.555
>3.25	2.17 (1.38-3.40)	0.001

Supplementary Figure 1: Flowchart of identification of individuals with NASH cirrhosis in the electronic health record.

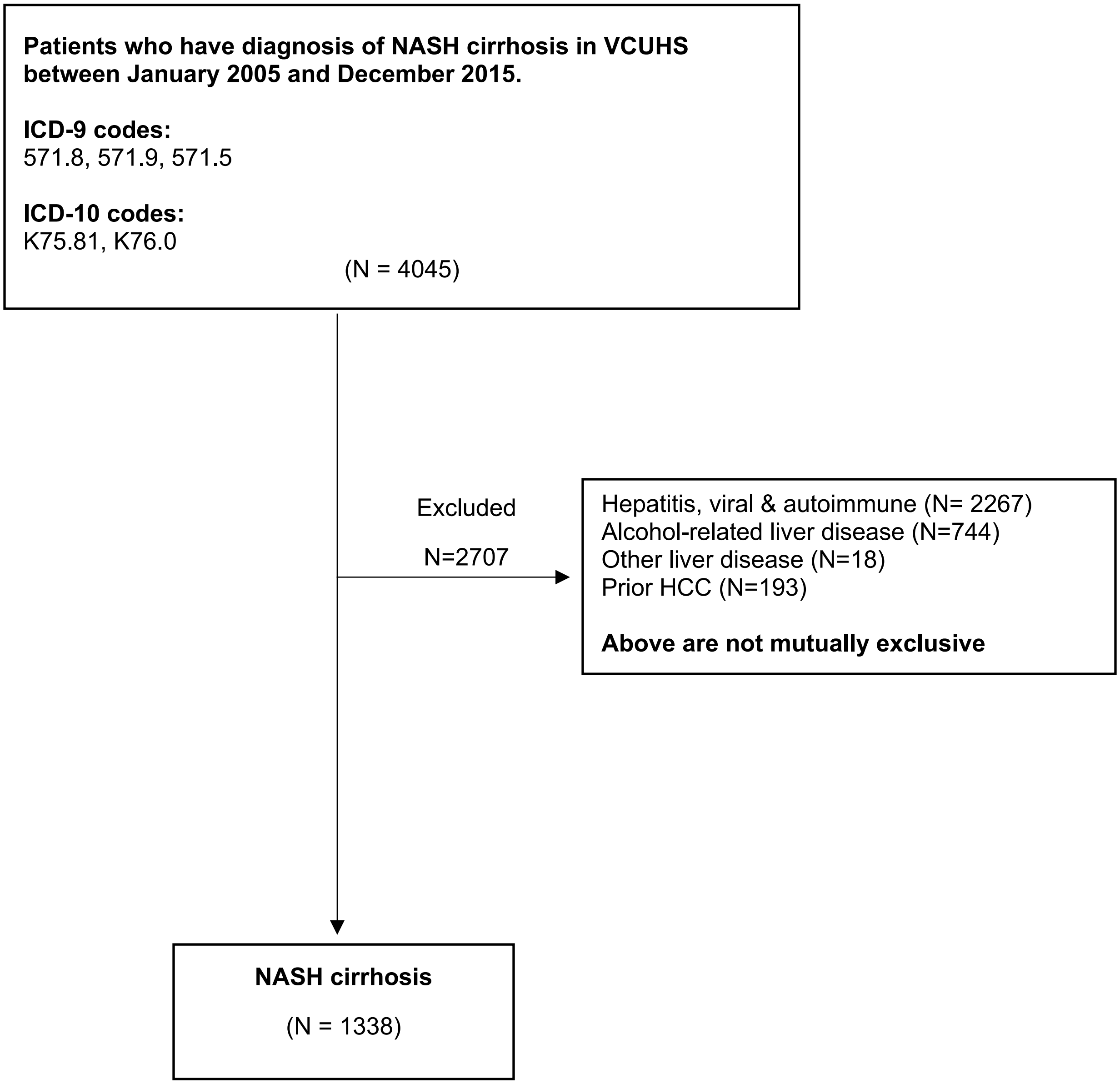

NASH, non-alcoholic steatohepatitis; VCUHS, Virginia Commonwealth University Health System; ICD, International Classification of Diseases; HCC, hepatocellular carcinoma.
